# Resolution of tongue lesions caused by *Leishmania infantum *in a dog treated with the association miltefosine-allopurinol

**DOI:** 10.1186/1756-3305-2-S1-S6

**Published:** 2009-03-26

**Authors:** Valentina Foglia Manzillo, Rosa Paparcone, Silvia Cappiello, Roberta De Santo, Paolo Bianciardi, Gaetano Oliva

**Affiliations:** 1 Department of Veterinary Clinical Science, Faculty of Veterinary Medicine, Naples, Italy; 2 Private Practitioner, Via dell'Usignolo, Milan, Italy

## Abstract

Canine leishmaniosis is a severe systemic disease caused by the kinetoplastid protozoan *Leishmania infantum*, an obligatory intracellular parasite of mammalian macrophages, transmitted by the bite of phlebotomine sandflies. The infection in dogs might occur without any clinical signs or might be characterised by chronic viscerocutaneous signs, such as lymphadenopathy, skin lesions, splenomegaly, onychogryphosis, and renal as well as ocular damage due to immunocomplex deposition. In atypical cases the parasites can be found in the striated musculature, the central nervous system, the endocrine glands or gonads, with or without functional damage. *Leishmania *infection might seldom induce oral lesions, particularly on the tongue. The authors describe the clinical case of a four-year old mongrel dog with tongue lesions caused by *L. infantum*. The dog was presented due to diarrhoea, lack of appetite and hypersalivation. Examination of the oral cavity revealed the presence of multiple red, nodular lesions on the dorsal and lateral surfaces of the tongue. Definite diagnosis of an infection with *L. infantum *was obtained by an indirect immunofluorescence antibody test (IFAT) and by the cytological identification of the parasite in nodular, lingual lesions and bone marrow aspirates. The dog was treated with a combination of miltefosine (Milteforan^®^, Virbac), 2 mg/kg orally once a day for four weeks and allopurinol (Ziloric^®^, GlaxoSmithKline), 10 mg/kg orally twice a day for six months. At the end of the treatment, the animal showed full remission of clinical signs. The authors outline the atypical manifestations in the oral cavity in combination with a *L. infantum *infection and discuss the therapeutic potential of the combination treatment of miltefosine and allopurinol in canine leishmaniosis.

## Findings

*L. infantum *is an obligatory intracellular parasite of mammalian macrophages transmitted by phlebotomine sandflies of the genus *Phlebotomus *(Old World) and *Lutzomyia *(New World). It is a heteroxenous parasite, *i.e*. it needs two hosts to develop. On the vertebrate side, the principal hosts of *L. infantum *are dogs and other members of the Canidae family (foxes, jackals, wolves). In Italy, *Phlebotomus perniciosus *is considered the main species responsible for the dispersion of the infection [1]. The disease is endemic in the Mediterranean basin, Asia and Latin America, but is also reported in a rising number of cases from non-endemic countries due to pet travel and import [2,3]. In Italy, CanL has been observed for long time only in southern, central and the insular regions [4]. Recently, there has been evidence that CanL is currently expanding to north-western Italy into areas with a continental climate, far away from the recognised endemic areas along the Mediterranean coast [5]. Parasite transmission occurs through the bite of an infected phlebotomine sandfly, although secondary modes of transmission (*e.g*. via blood transfusion and congenital transmission) have been suggested [6,7].

Clinically, CanL is characterised by chronic viscerocutaneous signs, such as lymphadenopathy, skin lesions (furfuraceus dermatitis, ulcers and nodular lesions), symmetrical alopecia [8], keratoconjunctivitis, epistaxis and diarrhoea [9]. In atypical cases the parasites can also be found in the striated musculature, the central nervous system and the endocrine glands or gonads, with or without functional damage [10]. Mucosal localizations are rarely described in dog [11-14]. All known anti-*Leishmania *drugs used for CanL therapy can lead to temporary or permanent remission of clinical signs, but none of them is sufficient to eradicate the infection from diseased dogs [15]. Nonetheless, chemotherapy does not guarantee parasitological healing, the cessation of infectivity to sandfly vectors feeding on the canine host and/or the prevention of clinical relapse. The most commonly used drugs against CanL are the parenteral pentavalent antimonial meglumine antimoniate and the oral purine analogue allopurinol. These two medications are frequently used in combination [16]. Pentavalent antimonials have to be administered for a period ranging between 4 and 8 weeks. Allopurinol is usually administered for several months after clinical stabilization has been obtained.

There are a number of reasons that provoke the decision to choose an alternative protocol to the first-line drug combination of meglumine antimoniate and allopurinol. Among others these are unresponsiveness to therapy, severe side effects, occurrence of frequent relapses, poor owner's compliance to mode and frequency of drug administration and the possible selection of drug resistant strains of *L. infantum*. Other drugs reported to have some efficacy against CanL include miltefosine, pentamidine, aminosidine (paromomycin), ketoconazole, metronidazole with spyramicin, marbofloxacin and domperidone. Howevermore extensive clinical studies are necessary to verify their therapeutic effectiveness. Very recently a combination between miltefosine and allopurinol was proposed as alternative protocol for the treatment of CanL [17].

**Figure 1:**
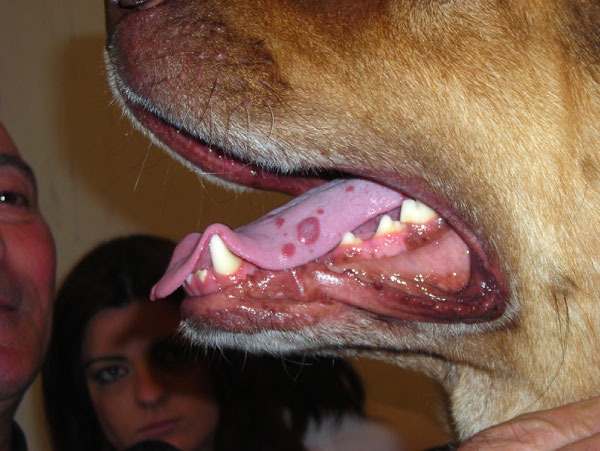
**Multiple red, nodular lesions on the dorsal and lateral surfaces of the tongue**.

In the following, a case report of a *L. infantum *infected dog with atypical lesions in the oral cavitiy, successfully treated with miltefosien and allopurinol is given. A four-year old mongrel dog was referred in March 2008 to the Department of Clinical Science, University of Naples (Italy), presenting a lack of appetite, diarrhoea and hypersalivation, together with the presence of multiple nodular lesions on the tongue. Clinical examination showed a slight lymphadenomegaly. Exploration of the oral cavity revealed the presence of multiple red, nodular lesions on the dorsal and lateral surfaces of the tongue (Figure 1). The following laboratory parameters were examined: complete blood count, total plasma protein, albumin/globulin ratio, urea, creatinine, alanine amino transferase (ALT) and complete urine analyses. All parameters were within the physiological range.

**Figure 2:**
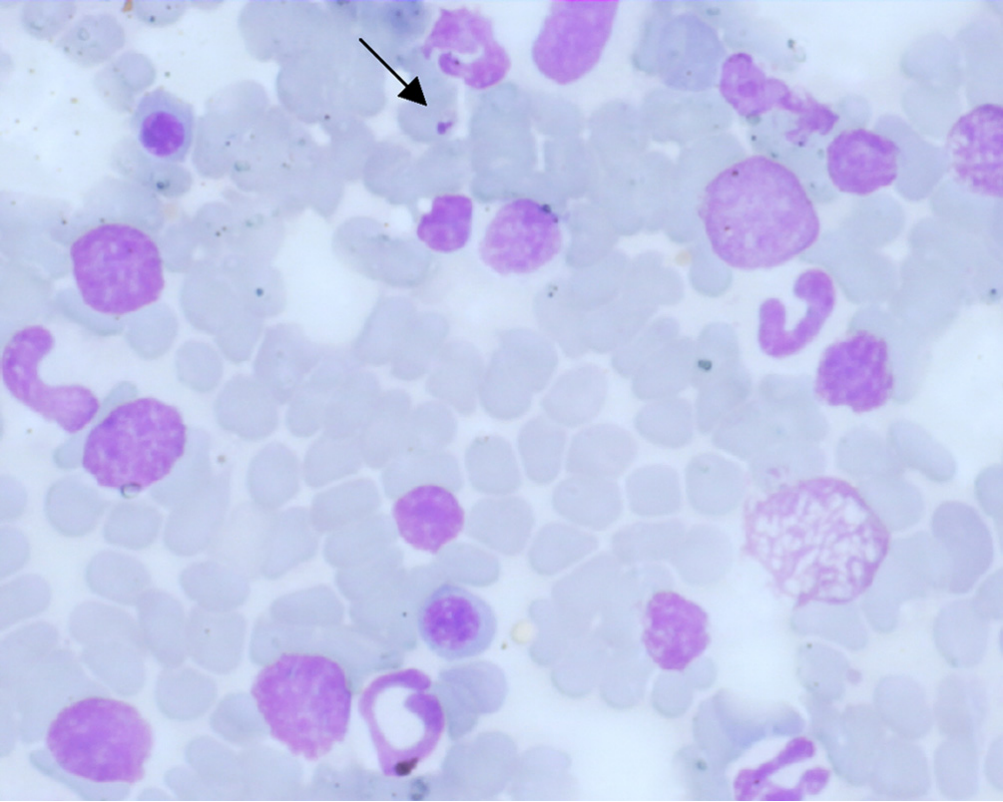
**Free amastigote form of Leishmania sp. in cytological material from the lingual lesions (May-Grünwald-Giemsa Quick; × 1000; bar = 25 μm)**. surfaces of the tongue.

Cytological examination of lingual nodular lesions demonstrated the presence of numerous neutrophils, macrophages, epithelial cells and lymphocytes. *Leishmania *amastigotes were detected within and outside macrophages (Figure 2). When examined by an indirect immunofluorescence antibody test (IFAT) for *L. infantum*, the dog was positive, showing a titre of 1:1280. Cytology of bone marrow-stained smears revealed a number of *Leishmania *amastigotes.

**Figure 3:**
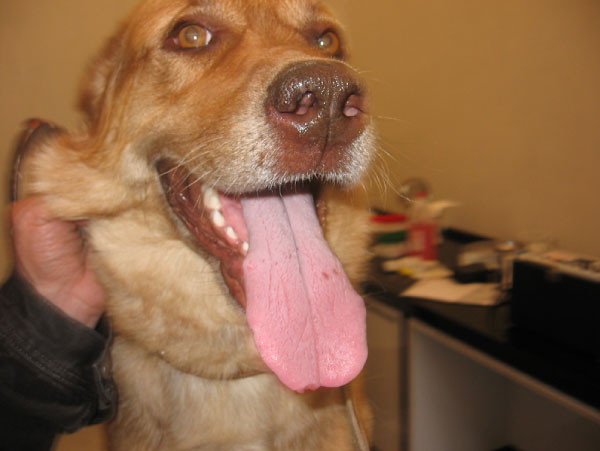
**Almost clinical resolution of the tongue lesions after treatment with a combination of miltefosine and allopurinol**.

The dog was treated with a combination of miltefosine (Milteforan^®^, Virbac), at a dosage of 2 mg/kg body weight orally once a day for four weeks and allopurinol (Ziloric^®^, GlaxoSmithKline), at 10 mg/kg body weight orally twice a day for six months. A good improvement of the systemic clinical signs was observed at the end of the miltefosine administration at four weeks, together with a partial resolution of the tongue lesions. The almost disappearance of the nodular tongue lesions was obtained after two months from the beginning of the therapy (Figure 3). Repeated haematological, biochemical and urine analyses proved again to be in the physiological range. The IFAT titre was recorded at 1:640. Cytological examination of tongue aspirates did not show any *Leishmania *amastigotes.

Zoonotic visceral leishmaniosis is the most widespread entity of zoonotic leishmaniosis caused by a single parasite species, *L. infantum*. Mucosal lesions caused by *L. infantum *are not often reported in dogs. In humans, oral lesions caused by *L. infantum *are not very common and usually observed in severely immunosuppressed patients (HIV co-infected patients, graft recipients [18]), though a case of an immunocompetent patient affected by leishmaniosis, characterized by a single lingual nodule and several palatine nodular lesions, has been described [19]. Oral *Leishmania *lesions, particularly on the tongue, are very rarely reported in dogs [12-14]. Tongue lesions in dogs are a probably the consequence of a systemic dissemination of the parasite [12]. Very rarely, it was also hypothesised that *Leishmania*-induced glossitis could represent a localised mucosal leishmaniosis, resulting from a new infection [13]. The above-described case confirms the possibility that *L. infantum *can be localised in the tongue tissue also in a dog without severe immunological depression. In this dog, due to the contemporary systemic involvement, showed by lymph node enlargement and bone marrow parasite dissemination, lingual lesions could probably result from an atypical dissemination of *Leishmania *parasites. The clinical condition determined the necessity of an anti-*Leishmania *drug therapy with leishmanicidal and systemic properties. Miltefosine, originally developed as an anti-cancer agent in humans [20] and registered for the treatment of human visceral leishmaniosis, has recently also been registered (Milteforan^®^) for the oral treatment of canine leishmaniosis in several European countries. The parasitological and clinical effectiveness of miltefosine has been documented in humans and rodents for many years [21,22]. With its direct anti-parasitic activity not depending on a functional immune system, its ease of use due to oral administration, and its low toxicity, miltefosine seems to meet all the requirements for a new drug to treat CanL. A potentially severe limitation for this oral agent could be the early development of drug resistance due to its long half-life [23]. To minimize the danger of generating resistance in *Leishmania *strains, it would be desirable to treat dogs and humans by different drugs, a suggestion hardly practicable with the drugs available at the moment.

Up to now, only few studies have been carried out in dogs with promising preliminary results. A recent study clearly demonstrated that miltefosine combined with allopurinol has clinical and parasitological effectiveness very similar to the combination of antimonials and allopurinol [17]. The association between these two drugs could have two beneficial effects, in terms of the potentiation of efficacy and the possible limitation of drug resistance. In the absence of definitive data about the new drug combination, our decision for this protocol was due to the easier oral administration of the two drugs. The complete clinical resolution of the above reported case and the absence of side effects confirm the safety and the promising efficacy of the combination between miltefosine and allopurinol for the treatment of CanL, even for the resolution of atypical localizations of the parasite.

## Competing interests

The authors declare that they have no competing interests.
